# Successful Complete Resection of Primary Cardiac Synovial Sarcoma Invading Right Atrium Wall

**DOI:** 10.1016/j.jaccas.2024.102706

**Published:** 2024-11-27

**Authors:** Kana Nagasawa, Takanori Kusuyama, Yuuya Tauchi, Yusuke Yamauchi, Zenichi Masuda, Naoto Inoue, Arata Hagikura, Takuma Onoe, Masato Komatsu, Hideya Mitsui

**Affiliations:** a Department of Cardiology, Tsukazaki Hospital, Himeji, Japan; b Department of Cardiovascular Surgery, Tsukazaki Hospital, Himeji, Japan; c Department of Oncology, Hyogo Cancer Center, Himeji, Japan; d Department of Diagnostic Pathology, Hyogo Cancer Center, Himeji, Japan

**Keywords:** acute heart failure, computed tomography, echocardiography

## Abstract

A 48-year-old man presented with several months of dyspnea and edema. He had no medical history. Transthoracic echocardiography showed a large mass in the tricuspid orifice. Enhanced computed tomography revealed that the mass had extended outside the heart by breaking though the right atrium wall. Hemodynamics has not collapsed yet, but his symptoms were rapidly worsening. He underwent emergency surgery, and complete resection was achieved. The pathologic diagnosis was primary cardiac synovial sarcoma. There were no signs of recurrence or metastasis 6 months after the surgery without additional treatment. Cardiac tumors are rare and develop silently, so they can be easily missed. Computed tomography and echocardiography provide much information quickly and help us to comprehend general condition. This case highlights their efficiency and the importance of early detection.

## Visual Summary

**Figure:**
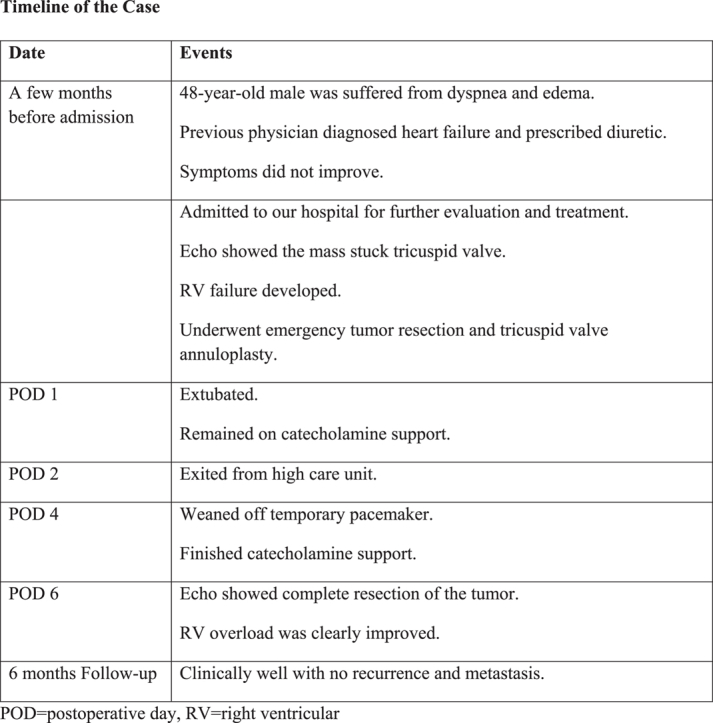

## History of Presentation

**Figure 1:**
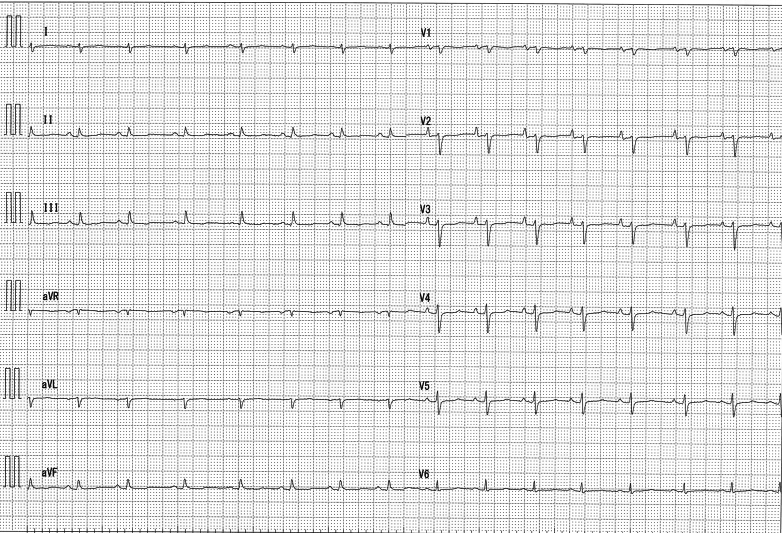
12-Lead Electrocardiogram R-wave is low in limb and chest leads. P-wave is high in anterior chest leads. This is evidence of right heart overload and pericardial effusion.

A 48-year-old previously healthy man, was suffering with dyspnea on effort and face and leg edema for several months. One month before admission, he had a medical examination at other hospital. Computed tomography (CT) showed pericardial and pleural effusion and ascites. The clinicians diagnosed congestive heart failure and prescribed diuretic. However, his symptoms did not improve. For further examination and treatment, he was referred to our hospital. At the time of his admission, his vital signs were as follows: temperature 96.8 °F, blood pressure 112/76 mm Hg, heart rate 89 beats/min regular, and oxygen saturation 98% (room air). Cardiopulmonary auscultation was normal. He had appreciable jugular venous distention and edema of face and the bilateral lower extremities. Electrocardiography showed sinus rhythm, atrial wave high in anterior chest leads, and low voltage in limb leads and chest leads (Figure 1). That meant right heart overload and increase of pericardial effusion. The QRS width was normal. There was no block. The admission diagnosis was right heart failure.

**Take-Home Messages**

• Cardiac tumors often progress asymptomatically but can cause life-threatening condition.
• Early detection with echocardiography and enhanced CT enables timely operation and life saving.

## Past Medical History

The patient’s medical history was significant for heart disease and no neoplasm.

## Differential Diagnosis

The differential diagnosis for the patient’s clinical situation included right heart failure, right valve disease, serositis, constrictive pericarditis, and masses with compressive effect on right atrium and ventricle. In this case, the symptoms were gradually worsening over several months. Tumors often progress slowly, so there was the possibility that a primary or metastasis cardiac tumor had disturbed flow in right heart with its growth.

## Investigations

**Figure 2:**
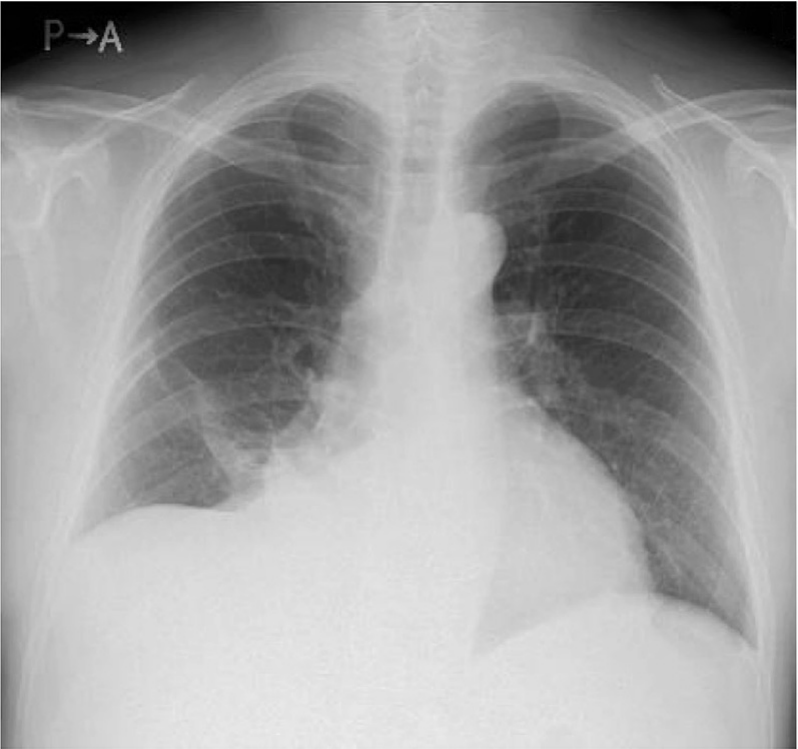
Chest X-Ray There is right slight pleural effusion and cardiomegaly.

**Figure 3:**
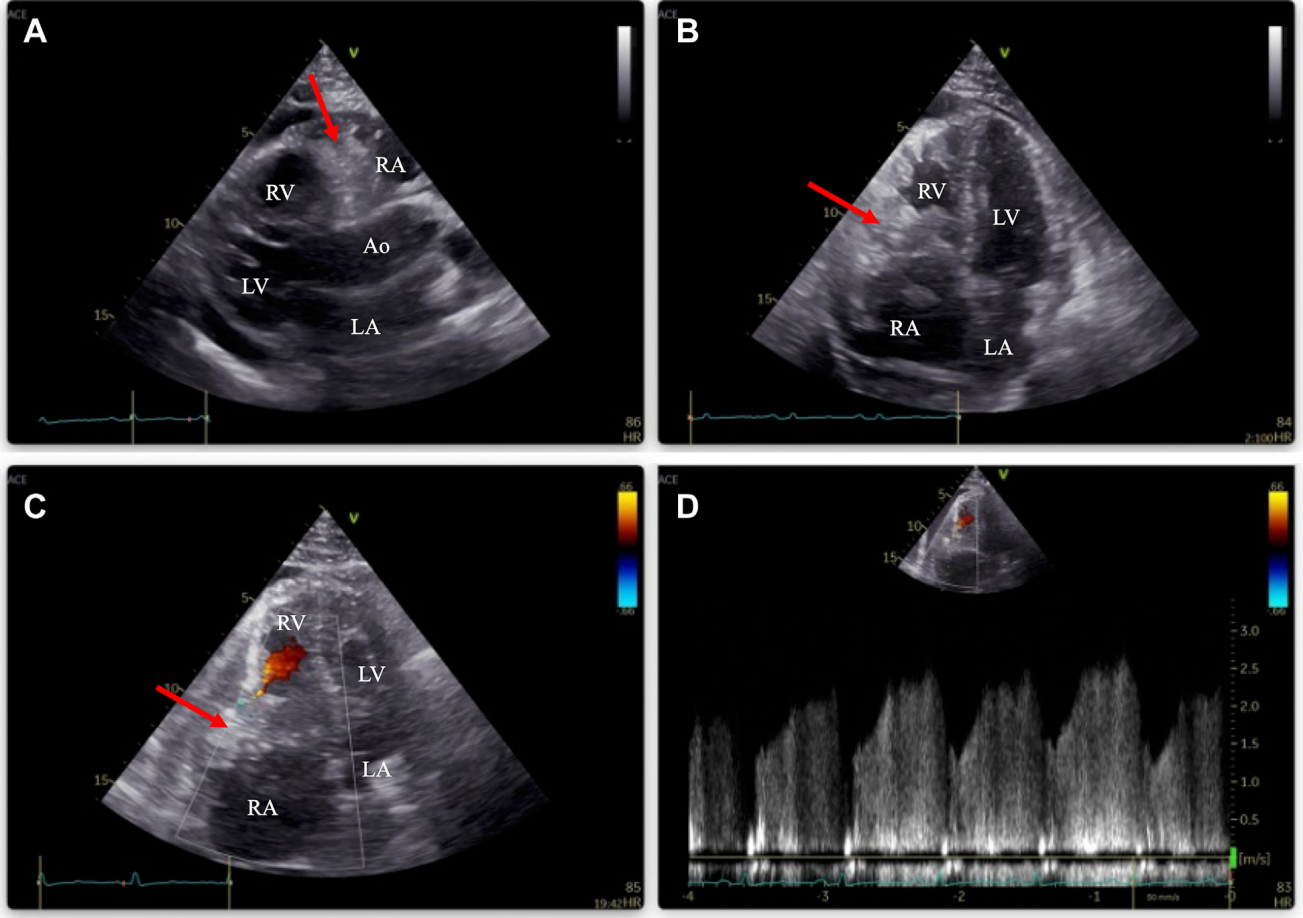
Transthoracic Echocardiography (A) Parasternal long-axis view and (B) 4-chamber view indicate that the mass (red arrows) is almost stuck in the tricuspid annulus. (C) Color Doppler image: Just a little inflow is observed. (D) Transtricuspid flow is continuous. The estimated diastolic pressure gradient of the tricuspid valve is 23 mm Hg. Ao = aorta; LA = left atrium; LV = left ventricle; RA = right atrium; RV = right ventricle.

**Figure 4:**
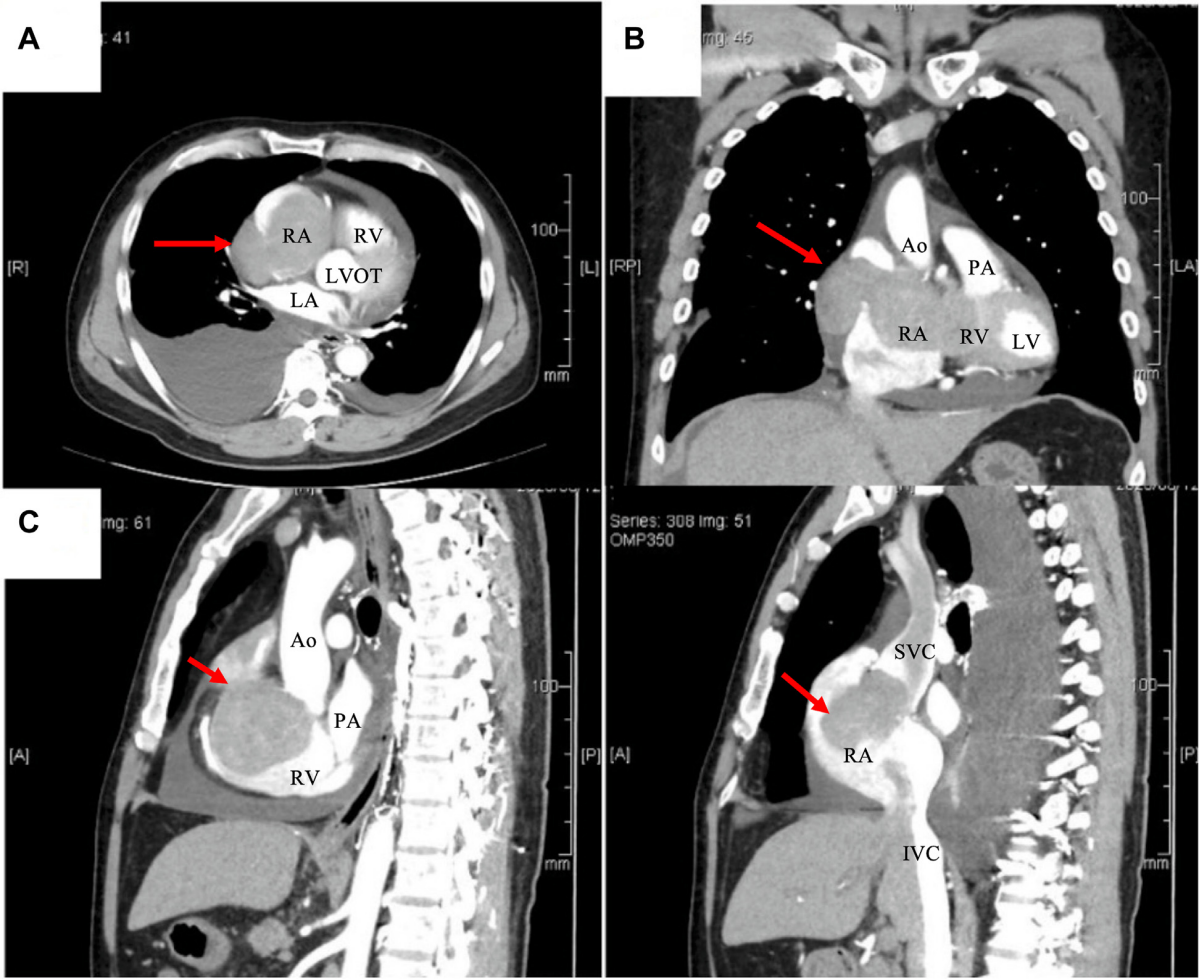
Enhanced Cardiac Computed Tomography (A) Axial view; (B) coronal view; (C) sagittal view. Enhanced computed tomography shows a 9.8 × 6.0 cm tumor mass (red arrows) and a large pericardial and pleural effusion. The mass extends outside of the heart, breaking though the right atrium free wall. It is smoothly marginated and isodense. Ao = aorta; IVC = inferior vena cava; LA = left atrium; LV = left ventricle; LVOT = left ventricular outflow tract; PA = pulmonary artery; RA = right atrium; RV = right ventricle; SVC = superior vena cava.

Chest X-ray showed cardiomegaly and right slightly pleural effusion (Figure 2). Laboratory examinations revealed congestive liver and renal dysfunction. Total bilirubin was 3.7 mg/dL, aspartate transaminase was 68 U/L, alanine transaminase was 48 U/L, and estimated glomerular filtration rate was 48.8 mL/min per 1.73 m^2^. B-type natriuretic peptide was 206 pg/mL, which also was abnormal. Other hematologic and biochemical analyses were normal. C-Reactive protein was 0.7 mg/dL. This was a little high but not significant. Tumor markers carcinoembryonic antigen and carbohydrate antigen 19-9 were 2.1 ng/mL and 11.2 U/mL, respectively, which were unremarkable. Transthoracic echocardiography showed a large mass almost obstructing the tricuspid orifice (Videos 1 and 2). Color Doppler images showed slight flow into the right ventricle. The color was moving from free wall to septum with heart beat, and the flow was observed in continuity. This meant that there was only a small residual flow though the tricuspid valve. The estimated diastolic pressure gradient of tricuspid valve was 23 mm Hg (Figure 3, Video 3). Pericardial effusion also was observed, with no signs of cardiac tamponade, and left ventricular function seemed to be normal. However, the quantitative evaluation revealed a decreased estimated cardiac output of 2.3 L/min and a decreased cardiac index of 1.2 L/min per m^2^. The patient underwent enhanced CT to disclose where the mass was placed, and the more detailed shape. It revealed that a large 9.8 × 6.0-cm mass extended outside of the heart by breaking through the right atrium free wall. There were no findings of invasion to superior vena cava or aorta. The mass was smoothly marginated and isodense (Figure 4). It was difficult to estimate the grade of malignancy in CT. Although magnetic resonance imaging and positron emission tomography (PET) are useful for tumor characterization, they need more time than CT. The patient condition was unstable, so we prioritized operation over image diagnosis.

## Management

**Figure 5:**
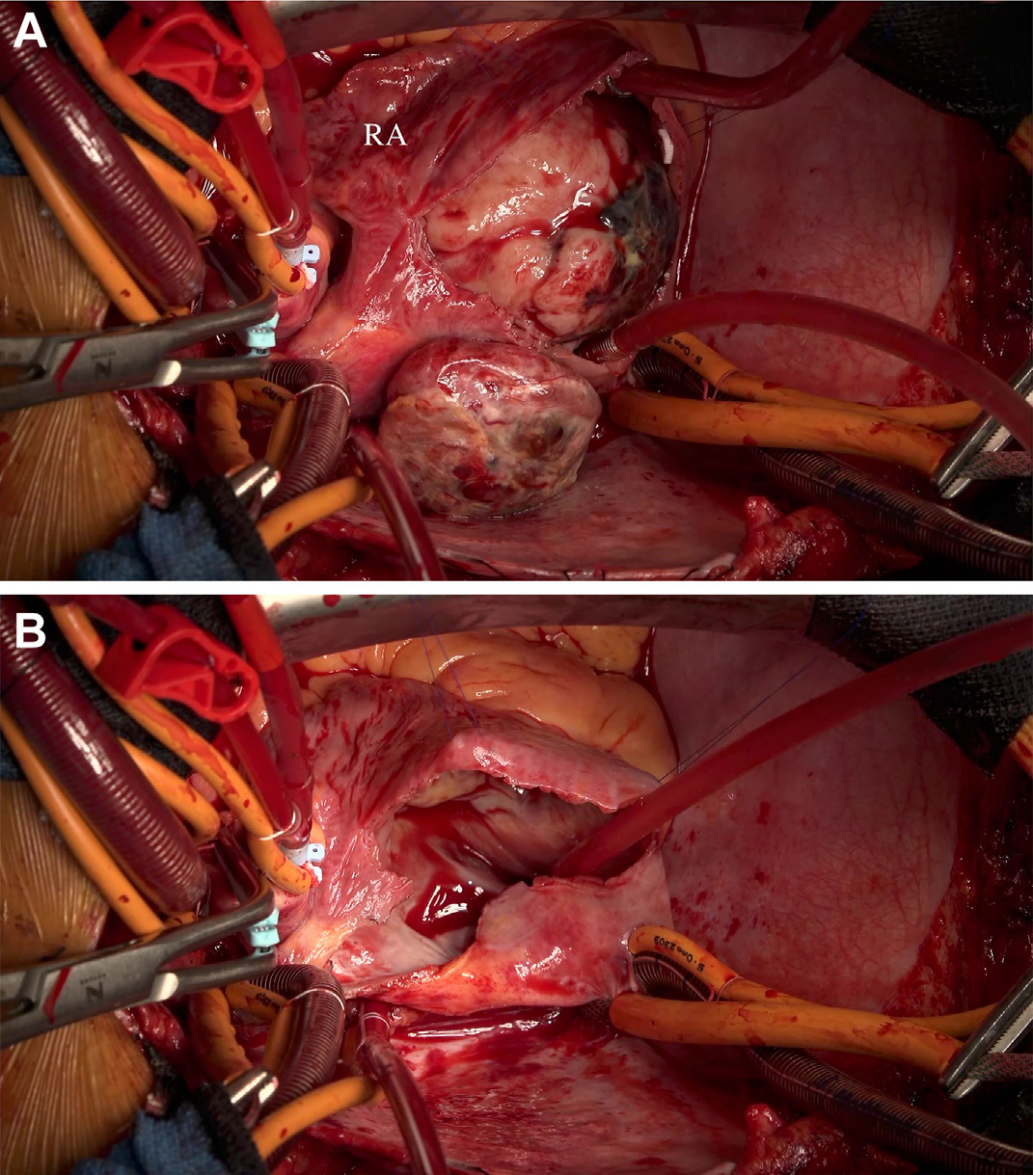
Intraoperative Views (A) Intraoperative view before tumor resection. A part of tumor extended extracardiac, breaking though the right atrium (RA) free wall. After right atrium incision, we recognized the tumor located directly below the RA wall and almost occupying right ventricle. (B) Intraoperative view after tumor resection. The tumor and the surrounding part of the RA free wall in contact with the tumor was resected, and the RA wall sutured directly. There were no macroscopic findings of tumor infiltration around tissue.

**Figure 6:**
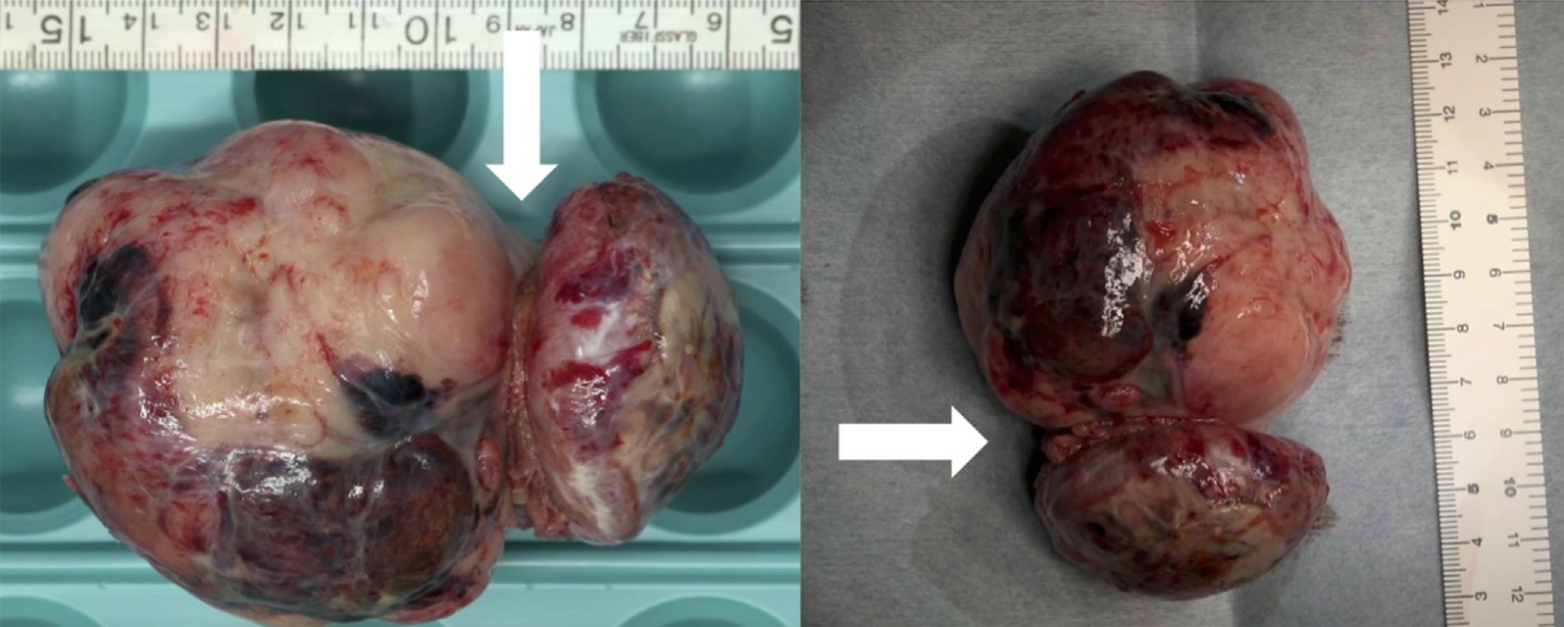
The Form and Size of the Specimen The specimen is a 9.0 × 6.0 cm mass. The tumor surface is smooth and there are white, red and black area mixed. The shaped part (white arrows) is the dividing point from inside to outside of right atrium.

**Figure 7:**
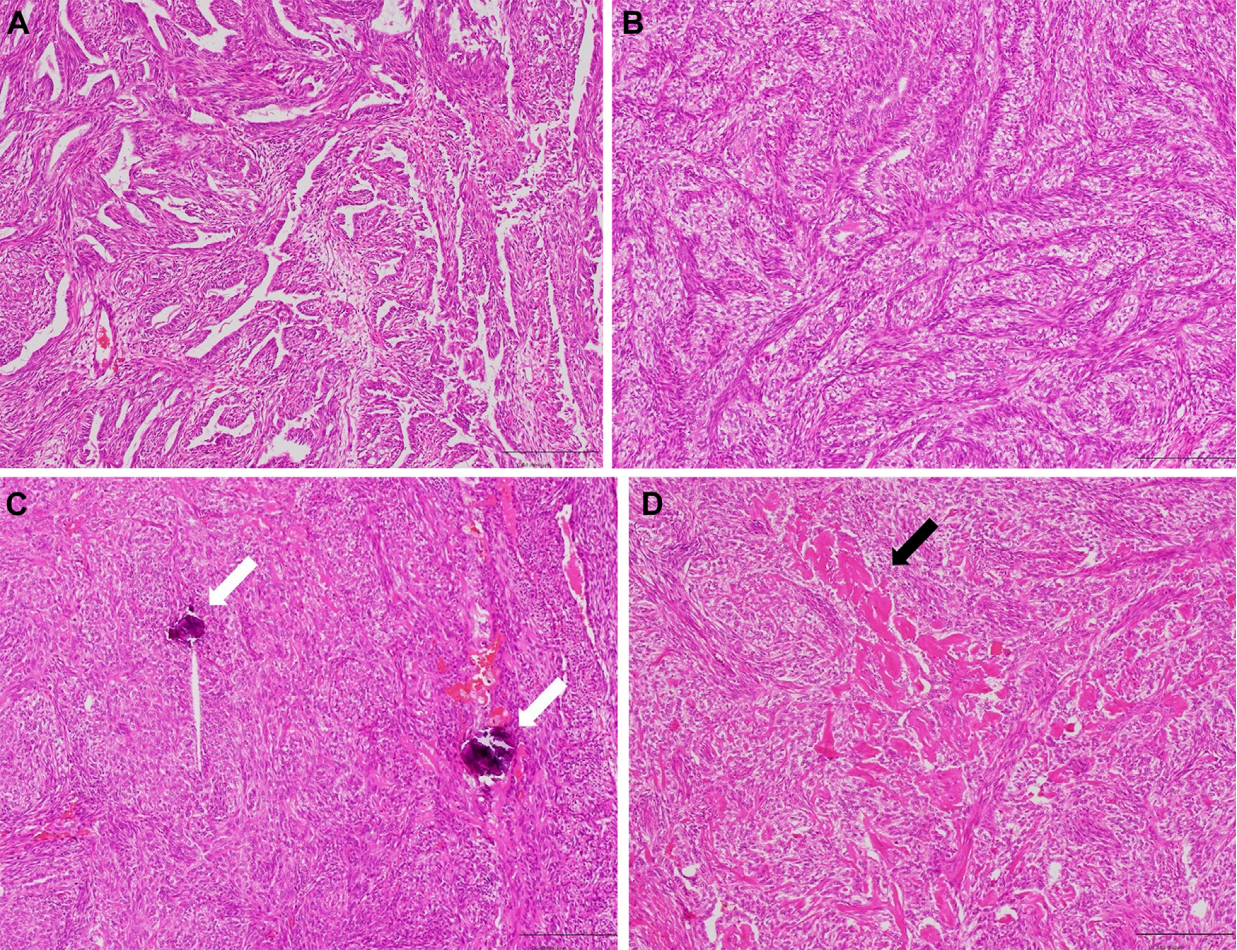
Histologic Analysis of the Specimen (A) The tumor consisted of spindle cell and epithelioid components of tubular and papillary material. (B) The cystoid and island-like epithelioid elements accounted for the most area. (C) Calcification (white arrows) was observed in some areas. This is a characteristic finding of synovial sarcoma. (D) Collagen fiber deposit (black arrow) was observed in some areas. This is a characteristic findings of synovial sarcoma. Hematoxylin and eosin stain, original magnification: ×100.

**Figure 8:**
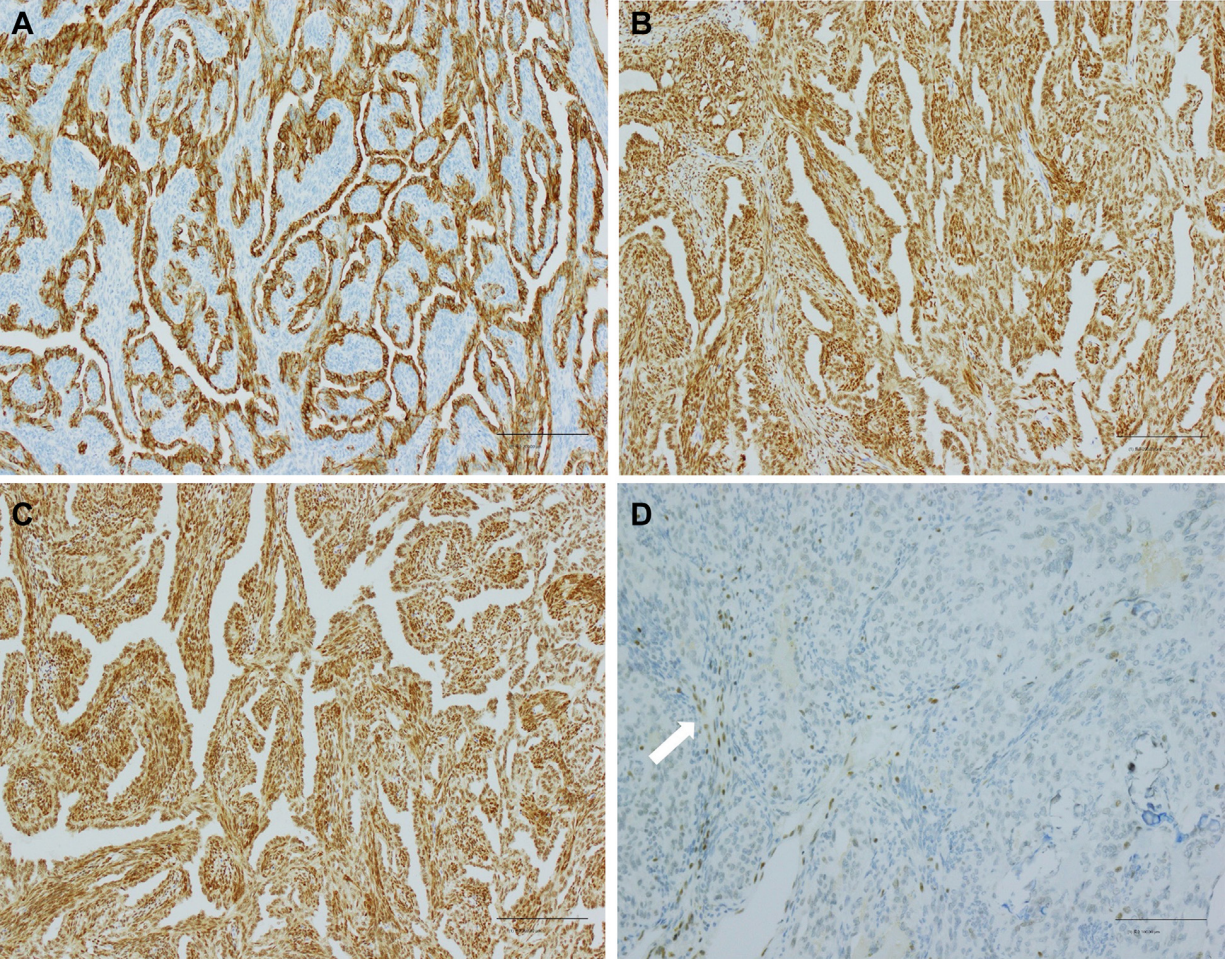
Immunohistochemical Analysis of the Specimen (A) Tubular epithelioid components are clearly immunopositive for AE1/3, and the spindle cells are negative (original magnification: ×100). (B) SS18-SX is immunopositive for both tubular epithelioid and spindle cells (original magnification: ×100). (C) SSX is immunopositive for both tubular epithelioid and spindle cells (original magnification: ×100). (D) SMARCB1 expression is definitely weak in tumor cells, whereas it expresses in normal cells (white arrow) (original magnification: ×200). This confirmed synovial sarcoma immunologic findings.

The mass was incarcerating tthe tricuspid valve, and his symptoms had rapidly deteriorated. Therefore, we decided to perform emergency surgery. Transesophageal echocardiography provided more detailed image (Videos 4 and 5). After median sternotomy and pericardiotomy, it was observed to be attached to the free wall of the right atrium, but there was no adhesion to the pericardium. Extracorporeal circulation was established at the same time. The incision of the right atrium revealed that the mass had invaded the right atrial free wall and partly obstructed the tricuspid orifice. At this time, it was impossible to estimate whether the mass infiltrated to the tricuspid valve. Proceeding tumor dissection revealed no infiltration macroscopically, so complete resection was achieved (Figure 5, Video 6). Rapid pathologic diagnosis revealed that the resected right heart wall margins were negative. After that, tricuspid valve annuloplasty was performed. The specimen was a 9.0 × 6.0 cm smooth-margin nodular mass (Figure 6). The pericardial fluid cytology was categorized as class II, and there were no malignant findings. Histologic examinations of the tumor revealed bunch growth of spindle-shaped cells among epithelioid cells presenting ductal structure. There was calcification and collagen fiber pigmentation in fibrosarcoma. A part of the tumor showed calcification and elastic fiber deposit (Figure 7). The immunohistochemistry results were positive for cytokeratin (AE1/3) in epithelioid and diffuse positive for cytokeratin (SS18-SSX, SSX) in both epithelial and spindle cells. (Figures 8A to 8C). The pathologic diagnosis was biphasic synovial sarcoma. SMARCB1 expression was weak compared with normal cells (Figure 8D), which conformed to immunologic characteristics of synovial sarcoma. Although the continuity between the right atrium and pericardium was not observed definitely, but the extra- and intracardiac parts of the tumor showed the same histologic findings. This suggests that the tumor originated from the right atrium.

## Outcome and Follow-Up

Only a few days of catecholamine support were needed, and the postoperative course went well. Transthoracic echocardiography indicated complete tumor resection and normal right ventricular function. Pericardial and pleural effusion clearly decreased, and the symptoms also improved. We referred him to a cancer specialty hospital after discharge. The oncologist decided not to administer postoperative chemotherapy for 2 reasons. First, there were no clear findings of metastasis. PET after surgery indicated no metastasis, and transthoracic echocardiography also showed no abnormalities in the heart. Second, there is no established treatment regimen for primary cardiac synovial sarcoma, and the possibility of side-effects was unclear, so observation without additional treatment was recommended. At 6 months after the surgery, he was cleared. PET also indicated no recurrence and metastasis.

## Discussion

Primary cardiac synovial sarcoma was first reported in 1978.^1^ Primary cardiac tumors are uncommon, occurring less frequently than metastatic tumors (in a ratio of approximately 1:20-40) and with an incidence in autopsy series ranging only from 0.001% to 0.003%.^2^, ^3^, ^4^ Most primary cardiac tumors are benign, with approximately 25% of them being malignant, the majority of which are sarcomas.^4^^,^^5^ Primary cardiac synovial sarcomas account for approximately only 4.2% of primary cardiac sarcomas.^4^^,^^6^ Synovial sarcomas tend to arise from the heart but can also arise from the pericardium. The predilection site is the right atrium, with a right-to-left ratio of 2:1.^7^ They are usually detected at an advanced stage, and most synovial sarcomas involve more than 1 anatomic compartment within the heart. The criterion standard for diagnosis is immunohistochemistry, but it is hard to achieve. This is because synovial sarcomas are often misdiagnosed or overlooked. As in our case, synovial sarcoma patients often present with symptoms of congestive heart failure, such as chest distress, shortness of breath, and edema. They are treated with drugs, such as diuretics. Medication can mask symptoms, so physicians do not do additional examination. Synovial sarcomas progress asymptomatically and cause a sudden collapse of hemodynamics. The boundary between symptomatic and asymptomatic is unclear, but symptoms are expected to appear when the tumor exceeds a certain size and affects hemodynamics. The critical tumor diameter depends on individual cardiac size, so it is uneasy to establish a reference. However, in the case of localized and small masses without adhesions, complete resection is possible and long-term survival is expected. Resection of small nonvalvular (<2.5 cm) or pericardial (<4.0 cm) synovial sarcomas allows survival beyond 3 years,^8^ but postsurgical survival is only 12-15 months with larger (>5.0 cm) intracardiac tumors.^6^

We found a few reports about primary cardiac synovial sarcoma with emergency surgery performed. One case was a primary synovial sarcoma of the right heart involving the tricuspid valve in an elderly Chinese woman. She underwent tumor resection surgery, and escaped death.^4^ Another case was one in which synovial sarcoma obstructed the right atrium and prolapsed into the right ventricle in a young Egyptian man. He also underwent emergency tumor resection, and it was successful. However, he succumbed to hemorrhage from a brain metastasis.^9^ The longest reported survival after surgery was 14 years. A combined modality therapy of surgery, chemotherapy, and radiotherapy was performed in that case.^10^ In our case, the tumor diameter was >5.0 cm and it was intracardiac. It is true that these were poor prognosis factors, but the tumor had not infiltrated yet. This is another important factor for success of the operation, so we suggest that the early diagnosis before infiltration contributes to complete resection, regardless of tumor size or location.

## Conclusions

Primary cardiac synovial sarcoma is a highly aggressive disease owing to its late presentation, although it should be noticed from imaging features. Complete resection can provide long-term survival, so the possibility of primary cardiac synovial sarcoma should be considered when we encounter unexplained heart failure. To make full use of imaging modalities, echocardiography and enhanced CT are useful for early detection and evaluation of hemodynamics. This enables prompt treatment decision, leading to life saving.

**Visual Summary:** Timeline of the Case

Date	Events
A few months before admission	48-year-old male was suffered from dyspnea and edema.
	Previous physician diagnosed heart failure and prescribed diuretic.
	Symptoms did not improve.
	Admitted to our hospital for further evaluation and treatment.
	Echo showed the mass stuck tricuspid valve.
	RV failure developed.
	Underwent emergency tumor resection and tricuspid valve annuloplasty.
POD 1	Extubated.
	Remained on catecholamine support.
POD 2	Exited from high care unit.
POD 4	Weaned off temporary pacemaker.
	Finished catecholamine support.
POD 6	Echo showed complete resection of the tumor.
	RV overload was clearly improved.
6 months follow-up	Clinically well with no recurrence and metastasis.

POD = postoperative day; RV = right ventricular.

## Funding Support and Author Disclosures

The authors have reported that they have no relationships relevant to the contents of this paper to disclose.

## Appendix

Video 1 Transthoracic Echocardiography: Parasternal Long-Axis View A part of the mass is observed in the right ventricle.

Video 2 Transthoracic Echocardiography: 4-Chamber View The mass appears wedged against the tricuspid valve.

Video 3 Color Doppler Image There is just a little inflow. It is slipping through a narrow space between the mass and tricuspid valve.

Video 4 Transesophageal Echocardiography: 4-Chamber View A part of the mass appears to be attached to the right atrial wall.

Video 5 Transesophageal Echocardiography: Right Atrium and Ventricle Focused View The mass echo brightness is uneven, suggesting malignancy.

Video 6 The Moment of Tumor Resection The tumor was removed smoothly as a mass by the surgeon’s hand.
